# Effect of human probiotics on memory, psychological and biological measures in elderly: A study protocol of bi-center, double-blind, randomized, placebo-controlled clinical trial (CleverAge Biota)

**DOI:** 10.3389/fnagi.2022.996234

**Published:** 2022-11-10

**Authors:** Ales Bartos, Josefina Weinerova, Sofia Diondet, Karel Vales

**Affiliations:** ^1^Third Faculty of Medicine, Charles University, Prague, Czechia; ^2^Department of Neurology, Faculty Hospital Královské Vinohrady, Prague, Czechia; ^3^National Institute of Mental Health, Prague, Czechia

**Keywords:** probiotic, psychological and biological measures, clinicaltrials.gov NCT05051501, memory, depression, clinical trial, gut microbiota, cognitive function

## Abstract

**Background:**

Gut microbiota may influence brain functions. Therefore, we prepared a study protocol for a double-blind, crossover, randomized clinical trial to determine the complex effects of human probiotics on memory, psychological, and biological measures in the elderly.

**Methods:**

We selected eligible participants using an effective electronic questionnaire containing the inclusion and exclusion criteria and a brief electronic cognitive test. One-third of the respondents with the worst cognitive scores on the electronic test are randomized to group A, starting with a 3-month probiotic intervention, and to group B, starting with a placebo. In a crossover design, both groups change their intervention/placebo status after 3 months for the next 3 months. Participants refusing longer personal assessments due to the COVID-19 pandemic were randomly allocated to one of two subgroups assessed online. Participants in both groups are matched in age, education, gender, and cognitive scores on electronic testing at baseline. At three time points, participants are assessed using a neuropsychological battery, self-report measures of mood, a physical fitness test, blood, urine, and stool samples, and actigraphy. A subset of participants also provided their biological samples and underwent the neuropsychological battery in an extended testing phase 3 months after study termination to find out the long-term effect of the intervention.

**Discussion:**

This is the first trial to address the comprehensive effects of human probiotics on memory and many other measures in the elderly. We assume that the probiotic group will have better outcomes than the placebo group after the first and second trimesters. We expect that the probiotic effect will persist for the next 3 months. These study’s findings will contribute to an interesting area of how to improve memory, psychological and biological and other factors naturally and will examine the importance of probiotics for overall health in the elderly.

**Clinical trial registration:**

[clinicaltrials.gov], identifier [NCT05051501].

## Introduction

The aging population may be associated with the worsening of memory and mood. Natural ways are sought to compensate for such impairments. One of the proposed options to achieve this is to modify the composition of gut microbiota and their by-products. This may be a surprising yet highly promising way to influence brain function and blood composition (Wilmanski et al., 2021; Wu et al., 2021; Li et al., 2022).

The gut microbiota is a vast bacterial community living in a symbiotic relationship with a human host. Neural, hormonal, and immunological signaling between the host and the microbiome occurs through the microbiota-gut-brain axis system (Collins et al., 2012; Wu et al., 2021; Li et al., 2022). The bidirectional nature of this communication axis has recently raised the question of whether memory impairment could be influenced by bolstering the gut microbiome.

The effects of microbiome interventions on changes in brain chemistry and cognition are a flourishing field within animal modeling (Desbonnet et al., 2008, 2010; Bercik et al., 2010). Notably, a recent study found that transplanting microbiota from young mice into older mice also attenuated aging-related cognitive impairment (Boehme et al., 2021). The intestinal microbiota in humans can have various effects on the brain (Collins et al., 2012). Gastrointestinal diseases, such as irritable bowel syndrome, commonly have psychiatric comorbidities (Wu, 2012). A clinical trial reported an increase in the mean Mini-Mental State Examination score by 1.9 points in patients with Alzheimer’s disease following a 12 weeks administration of probiotic supplements. The probiotic treatment had a favorable effect on some blood biomarkers (malondialdehyde, high sensitivity C-reactive protein, markers of insulin metabolism, and triglyceride levels) but not on other markers of oxidative stress and inflammation (Akbari et al., 2016).

The precise mechanisms through which microbiota can influence cognition and mood are not yet well-understood, with most evidence coming from animal models (Eastwood et al., 2021). Probiotics may affect brain function by influencing brain chemistry and neurotransmitter production (Eastwood et al., 2021; Salami, 2021). Bravo et al. (2011) have shown that probiotic supplementation in mice led to an altered expression of GABA receptors and also reduced anxious and depressive behavior. This effect was, however, only seen in mice with an intact vagus nerve (Bravo et al., 2011), suggesting that neural pathways also play a role in gut–brain communication. The gut microbiota can also influence brain function *via* immunological pathways. For example, the probiotic strains *Bifidobacteria* and *Lactobacilli* have been shown to reduce the production of proinflammatory cytokines (Desbonnet et al., 2008, 2010; Rodrigues et al., 2012) and increase the production of anti-inflammatory cytokines (Rodrigues et al., 2012). This may improve the blood-brain-barrier integrity and prevent bacterial translocation (Eastwood et al., 2021; Salami, 2021). In a study, administration of probiotics from strain Lactobacilli had a positive effect on memory performance in aged mice and, at the same time, reduced levels of inflammatory cytokines and increased the production of anti-inflammatory cytokines (Huang et al., 2018). Probiotics can also decrease oxidants and increase antioxidants (Salami, 2021). In a clinical trial with Alzheimer’s disease (AD) patients, Akbari et al. (2016) found that probiotic treatment decreased oxidant malondialdehyde and the inflammatory protein marker, high-sensitivity C-reactive protein. This was associated with an improved score on a memory test following the probiotic treatment.

Probiotics may be a promising option for improving common symptoms of several brain and psychiatric diseases. While the gut microbiota has been of great interest in animal models in recent years, evidence for its effect on memory, mood, or movement in humans is still scarce, as summarized by several recent reviews (Li et al., 2021; Salami, 2021; Klann et al., 2022; Mukilan, 2022; Xiang et al., 2022) or described in original papers (Marotta et al., 2019; Handajani et al., 2022). A probiotic supplement is responsible for memory retrieval in the case of memory impairment. Therefore, it could be of potential use in the treatment of neurodegenerative disorders such as mild cognitive impairment or Alzheimer’s disease (Mukilan, 2022). The interplay of gut microbiota and cognition has been the focus of recent research. Increasing evidence suggests that a healthy gut microbiota is crucial for normal cognitive processing. Some studies have focused on the effects of either gut microbiota or probiotic bacteria on the brain’s cognitive function in health and disease status (Salami, 2021). Indeed, the administration of tempeh-derived probiotics increased the cognitive domains of memory, language, and visuospatial function (Handajani et al., 2022). A study by Marotta et al. (2019) showed an improvement in depressive mood state, anger, and fatigue and improved sleep quality in the group using probiotics, while no between-group differences were found (Marotta et al., 2019).

A recent review summarized pre- and probiotic studies related to Parkinson’s disease in humans (Klann et al., 2022). They found out that human clinical trials investigating probiotic supplementation for treating or managing Parkinson’s disease are limited. Probiotics are thought to improve symptoms by altering the gut microbiota composition to reverse dysbiosis and disrupt inflammation-related pathways (Klann et al., 2022).

In the current paper, we introduce a randomized clinical trial protocol exploring the effects of novel probiotics from human supplements on memory, psychological and biochemical measures in older adults with normal cognitive performance and mild cognitive impairment. The study uses human probiotic supplements to see whether a 3-month treatment will improve selected measures in elderly participants. We extend previous research in several aspects. Our probiotics are obtained from the human microbiota. Currently, the vast majority of probiotic preparations come from beef cattle and are primarily used in the dairy industry. However, strains of bacteria from cattle and humans differ in many aspects. We use a randomized, double-blind, placebo-controlled clinical trial with a crossover design whereby participant groups both receive the treatment and the placebo but in the opposite order. This will allow us to compare outcomes both within and between groups. Additionally, in contrast to previously published literature, we (a) used multiple validated cognitive measures to monitor the treatment’s effect on cognition, (b) used various psychological and related aspects to evaluate the effect of the probiotic treatment on mood, (c) used multiple types of biological samples involving blood, urine, and stool samples to trace the effect of the treatment on biological measures, and (d) will explore a short-term effect of probiotics by repeated examinations 3 months after the study termination.

## Participants and methods

Our study was carried out at two centers: (1) the University Hospital Kralovske Vinohrady (UHKV), Charles University, Third Faculty of Medicine, Prague, and (2) the National Institute of Mental Health (NIMH) in Klecany near Prague, the Czech Republic, from January 2021 to May 2022. All the participants signed informed consent. The study was approved by the Ethics Committees of NIMH Klecany in 2020 (No 78 and 165/20) and UHKV Prague in 2020 (No EK-VP 17/0 and 1/2020). The study was registered on the clinicaltrials.gov portal with registration number NCT05051501.

### Inclusion and exclusion criteria of eligibility for the study

The inclusion criteria included age between 55 and 80 years, Czech as a native language, preservation of daily life activities within the community, and good sight and hearing.

Participants were excluded from the study if they had had any of the following diseases or conditions currently or in the past: a disease of the digestive tract (celiac disease, Crohn’s disease, etc.), a neurological disease of the brain (epilepsy, major head injury, stroke, brain operation, brain tumor, etc.), psychiatric disease or treatment (schizophrenia, bipolar disorder, drug addiction, alcoholism, etc.), organ failure (heart, kidney, etc.), an oncological disease in the last 5 years or were after chemotherapy or radiotherapy, an immune-mediated disease, an operation in general anesthesia in the previous 3 months, use of cognitive enhancers, and use of antibiotics or other probiotic supplements 3 months before the start of the study. Depression was not an exclusion criterion.

### Participant recruitment and allocation

We correctly expected that identifying appropriate people with many criteria would be difficult. Therefore, we chose a cost-effective way of recruitment using an electronic questionnaire with all the inclusion and exclusion criteria. People were invited to complete the electronic questionnaire *via* massive electronic advertisements on senior websites, local newspapers, and magazines, on television, on the radio, in newsletters, and advertisements distributed by the employers of investigators. Another source was contact databases from our previous projects (Bartos and Raisova, 2016; Bartos and Fayette, 2018; Bartos et al., 2020).

The second and major reason for electronic recruitment was an attempt to remotely assess episodic short-term memory and to choose those participants with mild memory impairment. Therefore, we have developed an electronic memory test with the abbreviation ALBAV (Bartos and Krejcova, 2022). The principles of this electronic examination were derived and modified from two validated tests for a brief cognitive evaluation in person, i.e., the Amnesia Light and Brief Assessment (ALBA) and the PICture Naming and Immediate Recall (PICNIR), introduced in the Czech neurological journal (Bartos, 2016, 2018, 2019; Bartos and Diondet, 2020). They were used in case reports on two patients with dementia and their adult children in English (Bartos, 2021).

Fully self-administered testing via the Internet consisted of three memory subtests. First, participants were asked to write down one correct name in ten consecutive black and white pictures. Then they wrote them down again in any order immediately after naming them. A number of correctly recalled picture names were recorded. Secondly, they were asked to copy and memorize a sentence of ten words. Almost every word belonged to a different grammatical case in the Czech language. Only delayed recall of correct words in the sentence was scored. Participants completed the third task between sentence encoding and delayed recall. They were instructed to carry out ten simple gestures described as infinitive verbs by one word on the screen. Participants were not instructed to memorize these gestures. After they had demonstrated all the gestures, they were asked to write down as many of the gesture verbs in any order as they could recall. The total score of the ALBAV was calculated as a sum of correctly recalled names of pictures, sentence words, and gestures, each with a 10-point maximum. Thus, the score ranged from 0 to 30 points. We invited only those individuals with possible memory impairment, as measured by ALBAV scores ≤20 points, to participate in our clinical trial. The purpose of these procedures was to effectively recruit potential participants with mild memory impairment and not cognitively normal individuals (it made no sense to improve their normal memory) and not patients with dementia (they were excluded with the inclusion criterion of preserved activities of daily living as stated in section “Inclusion and exclusion criteria of eligibility for the study”).

**FIGURE 1:**
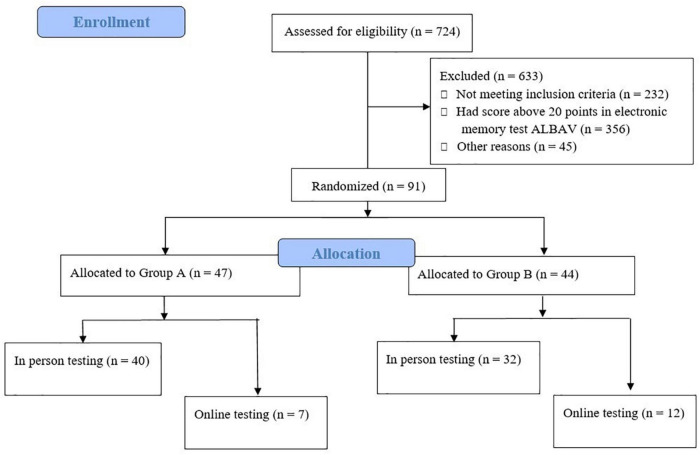
Participant recruitment and allocation flowchart.

The recruitment of participants is displayed in Figure 1. Hundreds of individuals completed the electronic questionnaire. Of those, 492 fulfilled all the criteria for participation in our study. Choosing only those participants with possible memory impairment using an ALBAV score ≤ 20 resulted in a further reduction of eligible participants to 136. The resulting number of participants represented 28% of the total, with the worst memory performance of the group that filled out the electronic recruitment questionnaire and the electronic memory test ALBAV and fulfilled all the criteria (*n* = 492). A final group of 91 participants was divided into two subgroups. A small subgroup (*n* = 19) of participants feared the COVID-19 infection during the pandemic. Thus, they were assessed with cognitive tests remotely *via* computers using monitors, web cameras, speakers, and microphones. Our experinces with distant testing were described recently (Polanska and Bartos, 2022). The rest of the procedures included completing self-report questionnaires, physical condition tests, and sample collection in person. All procedures were performed in person on the remaining 72 individuals.

### Trial design

**FIGURE 2:**
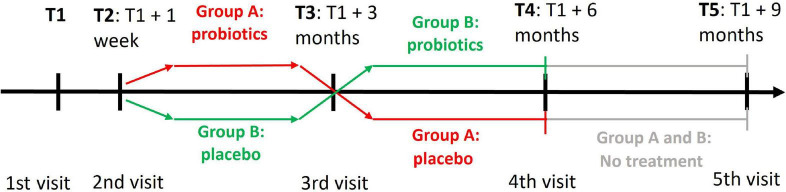
A cross-over design of the clinical trial with probiotics. T1 denotes the time of first visit when participants were assessed with the PROCOG cognitive battery. The two groups received both probiotic and placebo tablets but in a reverse order. At T2 the participants brought back baseline urine and stool samples and their blood was collected. They also got the first set of probiotic/placebo tablets. All examinations and procedures from T1 and T2 were repeated at T3 when the participants got the second set of probiotic/placebo tablets. All examinations and procedures similar to T3 were repeated at T4 and T5.

Our study was a randomized, double-blind, placebo-controlled trial with a crossover change of probiotics and placebo, which is shown in Figure 2.

All 91 participants were divided into two subgroups based on the order in which they completed the electronic registration form. One subgroup included participants with odd position numbers; the other one had even numbers. After checking for statistically significant sociodemographic differences between two subgroups, a few individuals were exchanged between these subgroups. This ensured that both subgroups matched in age, education, gender, and ALBAV scores before the start of the trial. Unexpectedly, 19 participants did not wish to come for testing in person due to the COVID-19 pandemic. Therefore, they were transferred from in-person to online assessment. These individuals were removed from the original allocation to online subgroups of participants.

A crossover design did not strictly confine one group of individuals to an intervention or placebo. Each group received both placebo and probiotics in tandem for 3 months each, but in reverse order. Members of group A start with a 3-month use of probiotics and continue with a 3-month use of placebo. The order of tablets is reversed for group B. There was no washout period between treatments.

Both participants and administrators were blinded to the type and order of intervention and placebo.

Each participant was evaluated during four visits at the University Hospital Kralovske Vinohrady, Prague, or the National Institute of Mental Health, Klecany, Czech Republic. A subset of the participants agreed with the last follow-up visit (*n* = 16 in group A, *n* = 18 in group B). They continued the study for a further 3 months without any intervention after the study termination to see the long-term effect of microbiome changes. Therefore, they had the fifth visit in addition. The first, third, fourth, and fifth visits were planned at 3-month intervals with a tolerance of ±1 week. The second visit was added 1 week after the first one to let participants take test tubes and bring back baseline stool and urine samples.

All measures were included at each visit except the first two. For logistical reasons, all procedures were divided into two visits at the beginning of the trial. Participants were administered a cognitive battery, completed self-report measures, and received test tubes to collect stool and urine samples during the first visit. The second visit included a physical fitness evaluation and sample collection. Participants also received either probiotic or placebo tablets and fiber or placebo fiber tablets for the next 3 months. A detailed description of each visit is described in Supplementary material 1.

Whenever possible, the administration of cognitive tests was conducted by the same administrator for individual participants throughout the whole period of the study until the fourth visit. Only two administrators performed cognitive testing on the fifth visit to each institution.

The participants received 3,000 Czech crowns for their participation at the end of the study (i.e., the fourth visit), which is equal to 123 EUR or 140 USD at the current currency exchange.

#### Online testing subgroups

A small subgroup of participants was afraid of COVID-19 infection during the pandemic. Thus, their cognitive testing was converted to an online examination to reduce personal contact time. The other data collection remained in person the same way as the rest of the participants because personal contacts were short. We wanted to have as many participants as possible with their measures: samples of biological material, questionnaires, physical fitness, and actigraphy.

### Probiotics and placebo

Participants were offered to take one probiotic/placebo tablet each day along with two fiber/placebo fiber tablets. The time of tablet consumption was up to the participant. More details about the content of the probiotic and placebo tablets are available in Supplementary material 2. All the tablets were provided by the company NEXARS (Brno, Czech Republic).

Participants were asked about the adverse side effects of the intervention at the end of each 3 months using a structured list of hypothetical side effects.

### Outcomes and measures

The effect of the probiotic intervention was evaluated using several comprehensive sets of cognitive tests, subjective questionnaires, visual scales, physical fitness, actigraphy, blood, urine, and stool samples.

#### Primary outcome measures

##### Cognitive measures

Cognitive function changes were assessed with brief tests and a neuropsychological battery, which took about 1 h on average. The reason was to show whether the influence of probiotics could be demonstrated with either approach or both. If proven, physicians and other professionals could utilize brief instruments in their practice to verify the favorable effect of probiotics. Seven raters were trained in all the tests by an experienced neuropsychologist. Each rater aimed to perform every cognitive evaluation of their participants for the whole clinical trial duration at all visits from the first to the fourth one to reduce assessment variability. Two versions of the cognitive battery were prepared, as participants were evaluated either in person or online. Firstly, we describe all tests for an in-person examination. Secondly, online versions of the tests follow and are explained. Table 1 shows a battery of tests and questionnaires administered in the same order at each testing session by the same rater throughout the clinical trial and their equivalents for online testing.

**TABLE 1:** List of tests and questionnaires administered at all visits in-person and online.

In-person order number	Test/questionnaire name	Online order number
1	The Clock Drawing test (CDT)	–
2	The Amnesia Light and Brief Assessment (ALBA) test	1
3	The Category (animal) fluency test	3
4	The Rey Auditory Verbal Learning Test (RAVLT) trials A1-A6	2
5	Trail Making Test (TMT) parts A and B	−
6	The Digit Symbol subtest from the Wechsler Adult Intelligence Scale (WAIS-III)	−
7	An anamnesis questionnaire	+
8	Short form of Geriatric Depression Scale (GDS)	+
9	The Beck Depression Inventory, 2nd version (BDI-II)	+
10	The Beck Anxiety Inventory (BAI)	+
11	The Functional Activities Questionnaire (FAQ-CZ)	+
12	The Questionnaire of Adverse Events	+
13	The RAVLT trial A7 delay recall	7
14	In-house cognitive battery ABACO including the PICNIR test	5
15	A questionnaire regarding side-effects of probiotics	+
16	The Dietary Habits Questionnaire	+
X	The phonemic fluency test (with initial letters NKP)	4
X	The Digit Span subtest from WAIS-III	6
		

ABACO, Assessment Battery of Cognition; PICNIR, Picture Naming and Immediate Recall. In-person order numbers are on the left, online order numbers are on the right. Three tests (−) were not administered online. All questionnaires were completed in-person (+).

##### Brief cognitive tests

Brief cognitive tests included the Clock Drawing Test (CDT), our newly developed the ALBA test, and a short cognitive battery (ABACO). The CDT was evaluated using our developed and validated scoring system called BaJa. It was weighted in favor of assessing clock hands and suppressing the contribution of the clock itself. It ranged from 0 to 5 points. The time of 23:20 was chosen as it is cognitively more difficult than 11:10 or 5:40, as proven in our report (Bartos et al., 2016). The CDT was used without modification in all testing sessions. The CDT was omitted in online testing.

The ALBA test is a newly developed three-minute examination of intentional and incidental short-term memory, which could differentiate patients with mild cognitive deficits from cognitively normal individuals. The innovative test paradigm was based on a recall combination of one short six-word sentence and six gestures. First, a participant was asked to remember a sentence. The following distraction included a demonstration of six gestures and their incidental and immediate recall. The final task was sentence recall (Bartos, 2019). Two alternate forms of the ALBA test are available with good comparable psychometric properties in the Czech Republic (Bartos and Diondet, 2020). Online ALBA was the same as the in-person one (Polanska and Bartos, 2022). A completed example of an ALBA form with detailed administration and evaluation instructions is shown in Supplementary Figure 1. The ALBA educational video is freely available at: https://www.youtube.com/watch?v=LyCuWc0-Gro.

Our in-house cognitive battery, ABACO, consists of five brief tests: (1) the Reading Encrypted Sentences subtest, (2) the sentence learning and recall, (3) verbal fluency test, (4) the PICture Naming and Immediate Recall (PICNIR), whose educational video is freely available at https://www.youtube.com/watch?v=cbJGtPG-nVA, and (5) the Five or Four-line test. Detailed information about them is in Supplementary material 3.

##### A comprehensive battery of neuropsychological tests

The Rey Auditory Verbal Learning Test (RAVLT) is intended for verbal memory evaluation. The test includes immediate verbal memory (A1), learning performance as a sum of five trials (A1–A5), proactive interference (B1), retroactive interference (A5–A6), and loss after consolidation (A6–A7) (Bean, 2011). In the Czech Republic, there are three alternative versions of RAVLT to eliminate the practice effect due to retest (Preiss et al., 2012). In the current study, we used them in version order: one at T1, two at T3, three at T4, and one at T5 (Figure 2). Online RAVLT testing was the same as that done in person.

The Trail Making Test (TMT) included parts A and B. Part A was used to evaluate processing speed, attention, and visuospatial ability. Part B, in addition to the mentioned functions, also assessed mental flexibility as a part of executive functions (Oosterman et al., 2010; Preiss et al., 2012). As there was no alternate form of the TMT in the Czech Republic, we used the same form for each test. The TMT was replaced with phonemic verbal fluency tests starting with the letters NKP during the online testing.

The Digit Symbol subtest from the Wechsler Adult Intelligence Scale III (WAIS-III) assessed the processing speed (Kaufman and Lichtenberger, 2005; Preiss et al., 2012).

Category (animal) fluency tests in person and online and phonemic fluency tests with initial letters NKP during online testing were used to evaluate executive functioning, language, and semantic memory (Lezak et al., 2012). Each verbal test took 1 min.

Online testing also included the Digit Span subtest from WAIS-III, which measured short-term verbal and working memory capacity (Preiss et al., 2012).

##### Self-report subjective questionnaires of depression and activities of daily living

Participants filled in the Geriatric Depression Scale (GDS) and the Functional Activities Questionnaire (FAQ-CZ) to assess their mood in the last 3 months and activities of daily living at each visit. The GDS ranged from 0 to 15 points, and FAQ-CZ ranged from 0 to 30 points. The more points, the more answers to depressive content in the GDS and the more impaired activities of daily living in the FAQ-CZ (Pfeffer et al., 1982; Lesher and Berryhill, 1994; Lach et al., 2010).

##### Biological surrogate markers of probiotic intervention

Serum, urine, and stool samples are taken for measurements of blood biochemistry, apolipoprotein E genotyping, metabolomic studies, and some brain-derived proteins and antibodies. The plan was to monitor metabolomics biomarkers, neurofilament light chain (NfL), and other measures and changes due to probiotic intervention. Participants provided their urine, fecal, and blood samples at each visit except the first one, as explained earlier.

#### Sample preparation

##### Serum sample collection and preparation

A nurse at each center took 13 ml of blood samples from participants who came for blood collection between seven and nine in the morning and who consumed their last meal the evening before. Blood was collected into two kinds of evacuated blood vacuum tubes (Vacuette 9 ml Serum and Vacuette 9 ml K3EDTA)—one for plasma and the second one for serum. After collection, the blood was immediately centrifuged at 4,000 rpm for 10 min, and the whole supernatant was transferred into a 5 ml Eppendorf tube, vortexed for 30 s, and divided into cryotubes for storage at -84^°^C till the analysis.

Before metabolomic High-Performance Liquid Chromatography Mass Spectrometry (HPLC-MS) analysis, one aliquot of serum (300 μl) thawed on ice, 600 μl of acetonitrile was added and vortexed for 30 s, and kept on ice for 10 min. Then, all samples were centrifuged at 13,500 rpm for 10 min at 4°C, and the liquid phase was transferred to vials for proper liquid chromatography-mass spectrometry (LC-MS) analysis.

##### Urine sample collection and preparation

A urine sample of 30 ml was collected on the same day in which study participants came for blood collection. We asked participants to take the first-morning urine for metabolomic purposes and consistency, specifically the second portion of their urine. They collected it into a sterile container, which they placed into a polystyrene transport box together with a freeze pack. When the participants bring their urine samples, they were centrifuged at 4,000 rpm for 10 min, then split into aliquots (500 μl) and stored at -84^°^C until the analysis.

Before metabolomic HPLC-MS/MS analysis, one aliquot of 500 μl urine was thawed on ice, 500 μl of acetonitrile was added, and it was vortexed for 30 s before being kept on ice for 10 min. Then, all samples were centrifuged at 13,500 rpm for 10 min at 4^°^C, and the liquid phase was transferred to vials for proper LC-MS analysis.

##### Stool sample collection and preparation

**FIGURE 3:**
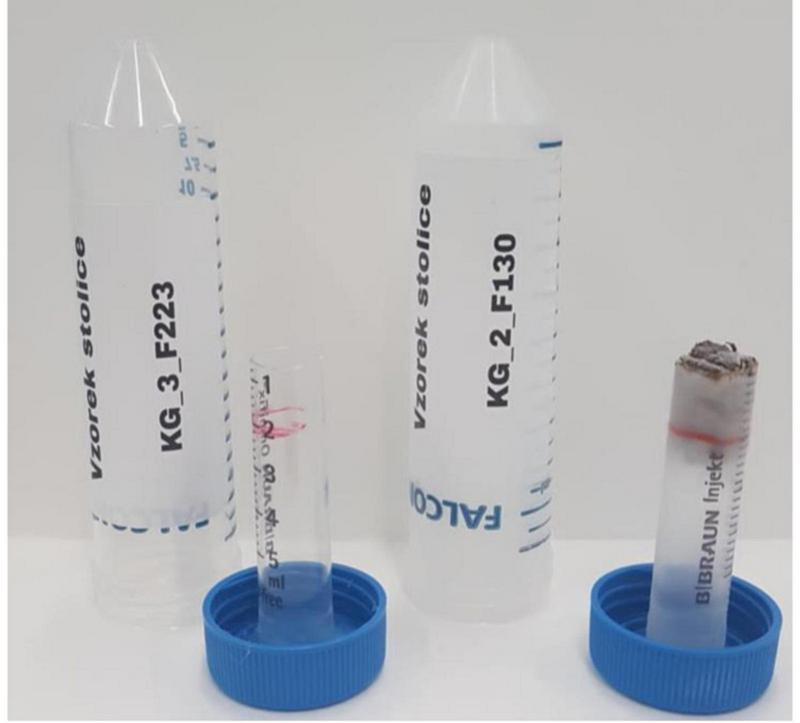
Special in-house tubes before (left) and after (right) collection of one 1.5–2 ml stool sample.

Each participant received a special toilet net during the first visit to avoid contact with stool and to prevent contamination from a toilet bowl. A participant took a stool sample of 1.5–2 ml from the central part of the participant’s stool immediately after defecation within 1 week before the planned visit. We provided them with special tubes we prepared in-house for this particular purpose. Our empty and filled tubes are shown in Figure 3. Stool samples were stored at -20^°^C in a home freezer. Participants brought them to the visit when the samples were stored at -84^°^C for proper long-term storage until the analysis.

Samples were thawed just before analysis. Stool pieces of a cherry stone size were transferred into polypropylene tubes, weighted, and the tubes were placed into a vacuum lyofilisator to dry all samples overnight. Each sample was weighed again, and a mixture of miliQ:MeOH: ACN (20:40:40) (v:v:v) was added in a ratio of 1:3 (stool weight: volume of mixture). Samples were placed into an ultrasound bath and sonicated for 20 min, followed by a 1 h incubation at 4°C. Finally, samples were centrifuged at 13,000 rpm for 10 min, and the supernatant was transferred into a vial for LC-MS analysis.

##### Sample preparation for quality control analysis of serum, urine, and stool

Quality control (QC) samples served to monitor the repeatability and stability of the HPLC-MS system during multiple analyses of serum, stool, and urine. QC samples were injected six times initially and then randomly injected after every five unknown samples.

One sample of 20 μl was prepared for QC analysis and stored together with other aliquots.

Stool samples of 5 μl of final supernatant from each sample are pipetted and mixed.

After thawing on ice, each sample was vortexed for 30 s, and then all the samples were mixed, and acetonitrile was added (v/v ratio of 1:2 for serum, 1:1 for urine). The final mixed sample was centrifuged at 13,500 rpm for 10 min, and the liquid phase was transferred into a vial for LC-MS analysis.

#### Conditions for high-performance liquid chromatography mass spectrometry measurements

Metabolomic analysis was performed on Ultimate 3,000 HPLC System (Thermo Fisher Scientific, USA), coupled with a quadrupole TOF detector—TripleTOF 5,600 (AB Sciex, Canada), in both positive and negative (ESI+, ESI-) modes with the IDA method. Samples were analyzed *via* separation on a Phenomenex HPLC Kinetex C18 2.6 μm 150 mm × 3 mm column (Phenomenex, USA) with SecurityGuard Column ultra performance liquid chromatography (UPLC). The column temperature is maintained at a constant 35^°^C. The mobile phase is composed of A = 0.1% formic acid in water and B = 0.1% formic acid in 100% acetonitrile for both positive and negative modes; the linear elution gradient from 5% B (0–2 min) to 100% B (18–23 min) was applied; the initial gradient conditions were restored within 2 min (23–25 min), and the last 5 min of the HPLC method (25–30 min) were applied to maintain the beginning conditions. The flow rates were 250 μL min^–1^, and the sample injection volume was 5 μl. Samples were held in an autosampler at 4^°^C, and each sample was injected twice for each m/z range (50–500 Da, 500–1,200 Da). The electrospray ionization (ESI) source conditions were set as follows: ion source gas one (GS1) 35, ion source gas two (GS2) 30, curtain gas 25, ion spray voltage 4,000 V, and source temperature 450^°^C.

#### Chemicals and reagents

Chromatographic-grade methanol, acetonitrile, and acetic acid were purchased from Honeywell (Riedel-de-Haen) and Sigma Chemical. Ultra-high purity water was produced by the Milipore-Q water purification system ELGA Purelab Flex three (VWS Deutschland, Germany).

#### Plasma neurofilament light chain

Plasma neurofilament light chains (NfL) were determined with commercially available ultrasensitive single molecule array technology (Quanterix, Billerica, MA) by team-certified laboratory technicians blinded to clinical data.

#### Secondary outcome measures

##### Self-report and subjective measures

###### Depression and anxiety questionnaires

The second version of the Beck Depression and Anxiety Inventory (BDI-II, BAI) was also used. Each questionnaire consisted of 21 items rated by participants on a scale ranging between 0–3 points, i.e., a total score between 0–63 points; the more points, the more depression or anxiety (Beck et al., 1988, 1996).

###### Subjective feelings using visual analog scales

Participants expressed their feelings about different aspects during the past 2 weeks using seven visual analog scales (VAS) with a range of 0–10 points. The following questions were to be answered: VAS one—memory: How would you rate your memory in the past 2 weeks? (0 = bad memory; 10 = excellent memory); VAS two—digestion: What were your feelings during eating, after eating, digestion, and bowel movements in the past 2 weeks? (0 = total discontentment; 10 = total contentment); VAS three—How did you feel overall regarding your health during the past 2 weeks? (0 = very bad health; 10 = excellent health); VAS four—sleep: What was the quality of your sleep during the past 2 weeks? (0 = bad sleep quality; 10 = excellent sleep quality); VAS five—the feeling of anxiety: How anxious were you during the past 2 weeks? (0 = no anxiety; 10 = maximum anxiety); VAS six—tiredness: How physically tired did you feel during the past 2 weeks? (0 = not at all; 10 = maximum tiredness); VAS seven—pain: How much pain did you have during the last 2 weeks? (0 = no pain; 10 = maximum pain).

###### Additional questionnaires

A Questionnaire of Adverse Events (QAE) consisted of 22 questions with regard to possible negative events in the past 3 months. Participants chose dichotomized answers yes/no. A total score was a sum of yes answers ranging between 0 and 22 points.

A questionnaire regarding the side effects of probiotics contained 11 questions about possible side effects of probiotic use. Participants chose from a binary yes/no scale whether they had experienced a particular side-effect during the past 3 months. The following side-effects were monitored: nausea, vomiting, constipation, flatulence, bloatedness, stomach cramps, excessive thirst, headache, insomnia, allergic skin reaction (itchiness, redness), and infection (urinary tract infection, pulmonary infection, etc.).

The Dietary Habits Questionnaire contained 20 items or questions about the participants’ eating and drinking, digestion, and other dietary habits (e.g., How often do you consume different food groups? How often do you consume different types of drinks? How much do you drink per day? What is the frequency of your stool?). Participants had multiple answers to choose from.

##### Measures of personal characteristics and physical fitness

Participants’ physical fitness was evaluated using the Beurer BF 950 diagnostic scale and a series of physical prowess tests. The scale provided these personal characteristics: weight, body mass index (BMI), body water percentage, muscle mass percentage, and bone weight.

Additionally, participants were asked about their past and current physical activities and their frequency. They completed three tests of physical performance. Firstly, we counted how many times participants fitted up one dumbbell of one kilogram from the extended arm to their shoulder in 30 s. Secondly, we measured the time it took them to walk a specific distance of 2 × 17 m in the corridor there and back. Thirdly, we counted how many times they stood up from a sitting position in a chair and back in 30 s.

## Proposed analysis

Data on the cognitive outcomes of the participants will be analyzed separately for groups evaluated in person and online. The results of online participants were exploratory due to the low sample size. Biochemical, self-report, and physical fitness outcomes were collected in person for all participants and were analyzed together. Statistical analyses will be conducted using R or Statistica.

Descriptive statistics will be calculated for primary and secondary outcomes at each visit.

The analysis will proceed using a between-subjects unpaired *t*-test at each time point or using a within-subjects paired *t*-test to compare results between time points.

Additionally, we will include apolipoprotein E allele status and its influence on outcomes.

### Power calculation

Given our sample size, we will able to detect the effect size of Cohen’s d = 0.43 in the matched-pairs two-tailed *t*-test, assuming power = 0.8 and α = 0.05. This will be d = 0.51 in those tests when only in-person data could be used.

For between-group comparisons, we could detect the effect size of Cohen’s d = 0.59 in a two-sample two-tailed *t*-test, assuming power = 0.8 and α = 0.05. This would be d = 0.67, assuming the use of only in-person data.

All power calculations were performed in G*Power 3.1.9.7 Software (Faul et al., 2007). As we did not attain equal group numbers, the sensitivity of the matched-pairs *t*-test is computed for the smaller group. This means we could detect a slightly smaller effect size for larger groups than reported here.

## Anticipated results and hypotheses

**FIGURE 4:**
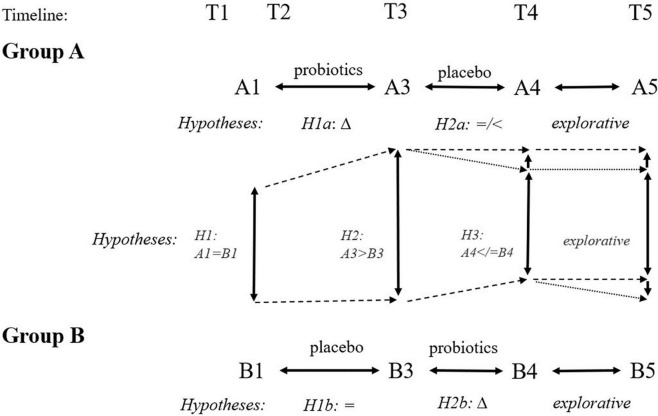
Expected hypotheses of cross-sectional (inter-group) and longitudinal (intra-group) comparisons at different time points. We expect improvement following the 3-month administration of probiotics (Δ: H1a, H2b, H2, H3). We expect no favorable change following the 3-month administration of placebo (H1b: =, H2a: =/<). Finally, we expect group B ending with the probiotic arm will have better outcomes than group A ending with placebo arm at time point T5 because of different intervals from the probiotic use (6 months for group A and 3 months for group B).

Our hypotheses are visualized in Figure 4. We expected a logical and favorable change in the cognitive tests, psychological measures, surrogate biological markers, and other measures following the 3-month probiotic treatment. Participants with at least one apoE four alleles would have worse results and, therefore, may benefit from probiotics more.

## Discussion

We prepared the first 6-month clinical trial to explore the effect of human probiotics in individuals with possible memory deficits. The aim was to include younger elderly individuals between 55 and 80 years of age for several reasons. This population is more prevalent and has a higher frequency of memory impairment than younger people. In addition, they are at the right age for memory improvement and interventions before they get too old or develop dementia.

We believe that the best target population includes individuals who are neither cognitively healthy nor suffering from dementia. On the one hand, healthy cognitive status is not necessary to improve. It may result in ceiling results without being able to observe a change after any intervention. On the other hand, patients with dementia have an advanced pathological processes in their brains. Managing their cooperation in complex studies of this kind is likely logistically costly or virtually impossible (e.g., taking stool samples in a standard manner to special test tubes within a certain time), and their comorbidities might affect the resulting data. Thus, individuals with mild memory deficits are ideal candidates for studies of probiotic effects. They need to improve their memory, and a positive change in test scores and other measures could be seen. However, it is problematic to find such individuals, motivate them to cooperate long-term, and achieve sufficient participant numbers after the exclusion of those with confounding factors (brain, psychiatric, and gastrointestinal diseases, among other conditions). This is highlighted by the fact that 724 individuals interested in our probiotic study using the electronic questionnaires were reduced to 91 (13%) eligible participants only, i.e., a ratio of eight interested persons to one included person in the study.

We chose an intervention using probiotics and prebiotics. A relationship between the gut microbiota and brain functions is considered in several diseases or conditions (Marotta et al., 2019; Salami, 2021; Handajani et al., 2022; Klann et al., 2022; Mukilan, 2022; Xiang et al., 2022). If proven effective, this natural way would be an easily feasible approach to prevent or postpone more pronounced memory decline or improve mood or anxiety in the elderly. In our comprehensive study, we decided to explore different aspects of brain functioning, physical performance, and blood, urine, and stool changes in response to probiotics with prebiotics. We use an explorative approach to determine which measures are influenced due to the intervention. We chose a crossover study design to ensure active intervention for each participant, which may achieve higher compliance and allow us to use each participant as a control for their own scores.

Probiotic treatment was intended for 3 months. A longer interval would prolong the duration of the study, make the trial more logistically complicated, and decrease participants’ compliance with the study. Three months of treatment was used in another related study (Handajani et al., 2022). The duration of our grant support also limits us. Our study was extended to 9 months for additional examinations to see the long-term effect of the intervention in a subset of participants.

The number of assessments and two types of tablets with a change after 3 months required careful management and sufficient and different personal resources and skills, facility equipment, and expert experience in many disciplines. We balanced recruitment with examinations and space capacities. This was the reason for establishing two centers for performing the study and limiting the number of participants included. Unfortunately, the COVID-19 pandemic complicated such a comprehensive clinical trial. Some participants refused to come to in-person assessments. The online testing had to be prepared for a relatively small group. Changing personal assessments to online ones might be an innovative approach to testing if a positive signal will be proven even in our small subgroups.

Our clinical trial has several strengths and limitations. First, we prepared a long list of inclusion and exclusion criteria to select appropriate candidates without present or previous brain, neurological, psychiatric, or gastrointestinal diseases and additionally fulfilling other conditions (participation at regular intervals for 6 months, appropriate veins, self-collection of stool and urine, etc.). Second, we created time-efficient recruitment using the electronic questionnaire for data collection from interested volunteers. Third, their memory was evaluated using an electronic test to invite only those with the worst results. This effective type of preselection saves resources and ensures a relatively homogenous group. Fourth, another strength is the comprehensive evaluation of probiotics with many cognitive tests, questionnaires, visual scales, psychological measures, and biological samples involving unusual materials like stool and urine. Fifth, an advantage is the use of probiotics of human origin. Moreover, we add prebiotics to stimulate bacterial growth.

A limitation includes a highly selected study population due to a high number of eligible criteria. The reason was to reduce confounding factors during the trial. Thus, our conclusions cannot be generalized to patients with mild cognitive impairment or deficits. This group of patients should be the next target of a probiotic intervention, although their comorbidities might change outcomes.

In conclusion, we wonder whether manipulating gut microbiota *via* probiotics will favor health conditions in the elderly. This study aims to contribute to this attractive idea in line with the contemporary high interest in interactions between the brain and microbiota.

## Ethics statement

The studies involving human participants were reviewed and approved by Ethics Committee of the National Institute of Mental Health, Klecany and the Ethics Committee of the University Hospital Královské Vinohrady, Prague. The patients/participants provided their written informed consent to participate in this study.

## Author contributions

AB: conceptualization, the data analysis plan, writing and revisions of an original manucript, and funding acquisition. JW and SD: writing and revisions of the manuscript and the data analysis plan. KV: conceptualization, funding acquisition, and a review and editing. All authors contributed to the article and approved the final manuscript.
