# Supplementary material for: Effect of human probiotics on memory, psychological and biological measures in elderly: A study protocol of bi-center, double-blind, randomized, placebo-controlled clinical trial (CleverAge Biota)
Source: Front Aging Neurosci. 2022 Nov 10;14:996234. doi: 10.3389/fnagi.2022.996234 (PMC9686296; doi:10.3389/fnagi.2022.996234)
Supplement: Supplementary file 1 [file Data_Sheet_1.docx]

**Supplementary Material 1: Neuropsychological measures and a detailed description of each of visit**

On the 1^st^ testing session the participants were tested by the PROCOG cognitive test battery, self-report questionnaires on depression, anxiety, unpleasant past events, current mood, health problems (specifically focused on health of the digestive tract) and dietary habits (full list of measures and questionnaires to be used and their properties is described in the Measures section). The administrator then scheduled the following 3^rd^ and 4^th^ visit in 3-months and 6-months’ time. The participant also received instructions and sample tubes for the collection and transport of urine and stool samples.

The 2^nd^ testing session takes place a week from the first testing. Participants brought the test-tubes with urine and stool samples and a nurse extracts blood samples. Afterwards, participants were tested on their physical fitness.

On the 3^rd^ testing session, participants first provided biological samples as per 2^nd^ testing session and then were tested again by the same measures as on the 1^st^ testing session. When possible, different versions of the measures are administered (all tests administered are described below in the Measures section).

The 4^th^ testing session was the same as the 3^rd^ testing session. Participants scheduled date of the next visit with the administrator at the end. Since the 5^th^ visit was planned after the end of recruitment and participants’ have originally been advertised only the 4 previous visits, they received the reward for study participation (3 000 CZK) at the end of the 4^th^ visit. Their participation in the 5^th^ visit was strictly voluntary and receiving the reward was not dependent on their choice of continuing or not-continuing into the 5^th^ visit.

The 5^th^ testing session took place again after 3-months interval. The course of the session is the same as 3^rd^ and 4^th^ testing session.
