# Supplementary material for: Effect of human probiotics on memory, psychological and biological measures in elderly: A study protocol of bi-center, double-blind, randomized, placebo-controlled clinical trial (CleverAge Biota)
Source: Front Aging Neurosci. 2022 Nov 10;14:996234. doi: 10.3389/fnagi.2022.996234 (PMC9686296; doi:10.3389/fnagi.2022.996234)
Supplement: Supplementary file 2 [file Data_Sheet_2.docx]

**Supplementary Material 2: Content of the probiotics and placebo**

Unlike other products available on the market, which are usually of bovine origin, our probiotics are manufactured using human-stemmed lines from a database of company NEXARS with an immunomodulatory effect demonstrated by a reduction in CD4-IFNγ in spleen of the tested mice. They contain bacterial strains of Streptococcus thermophilus GH, Streptococcus salivarius GH NEXARS, Lactobacilus plantarum GH and Pediococcus pentosaceus GH. Company NEXARS performed whole genome sequencing in all selected probiotic strains to exclude transmission bacterial resistance which is necessary from a safety point of view. Streptococcus salivarius GH NEXARS is listed in the Czech Collection of Microorganisms under code CCM 8797. Streptococcus salivarius GH NEXARS File no. PATB 11/18 is an original biotechnological production strain internationally protected under the patent procedure according to Budapest Treaty. The sequenced genome is available in the GenBank database at https://www.ncbi.nlm.nih.gov/bioproject/670337. The second set of fiber tablets contain micronized apple fiber to facilitate growth of probiotics.

The placebo for probiotics was composed of semi-coarse wheat flour, starch, maltodextrin and magnesium stearate. The fiber placebo was composed of cellulose, maltodextrin and stearate magnesium. The placebo tablets were visually and tastefully identical to the probiotic tablets.
