# Supplementary material for: Effect of human probiotics on memory, psychological and biological measures in elderly: A study protocol of bi-center, double-blind, randomized, placebo-controlled clinical trial (CleverAge Biota)
Source: Front Aging Neurosci. 2022 Nov 10;14:996234. doi: 10.3389/fnagi.2022.996234 (PMC9686296; doi:10.3389/fnagi.2022.996234)
Supplement: Supplementary file 3 [file Data_Sheet_3.docx]

**Supplementary Material 3: Description of our in-house cognitive battery the Assessment BAttery of COgnition (ABACO )**

The **A**ssessment **BA**ttery of **CO**gnition is abbreviated from the initial letters title as ABACO. It starts with the Reading Encrypted Sentences subtest that focuses on visual attention, discrimination and meaning comprehension. The aim of the examinee is to decipher three encrypted sentences. An example is: “W3 W1SH Y0U 4 PL34S4NT D4Y” which should be correctly read as “We wish you a pleasant day”. There are two parallel versions of this subtest and their reliability was proven in Holla’s master’s thesis (Hollá & Bartoš, 2017). In online testing an administrator shared a screen with the same encrypted sentences to examinee.

The next task was sentence learning and recall. It evaluates short-term verbal memory. It consisted of four parts. At the beginning an examinee repeated and learned the sentence of 10 words after two readings by the examiner. The English translation of the sentence is: “Seven old friends briskly walk along a narrow path through a dense forest”.

Another brief subtest of the fruit or profession verbal fluency served as distraction after which an immediate recall of the sentence followed. The last part was the delayed recall of the sentence after next two tasks. There are two almost parallel versions of this subtest with a difference of a half word on average (Hollá & Bartoš, 2017). Distraction was the verbal fluency test with category of fruits or jobs in parallel version 2 during 30 seconds (Bartoš & Raisová, 2019). A next task was the PICture Naming and Immediate Recall (PICNIR) whose filled form is shown in Supplementary Figure 2. The PICNIR educational video is freely available at <https://youtu.be/cbJGtPG-nVA>. The test has two parts. First, the examinees are to write a name under each of the 20 pictures in one word and remember these picture names. Second, without distraction, they are to recall and write as many picture names as possible during one minute. This test assesses written language, long-term semantic and short-term memory (Bartoš, 2016, Bartoš, 2018). There are two parallel and proven equivalent versions of this subtest (Hollá & Bartoš, 2017). In online testing this subtest was adapted to a verbal version. The examinees name pictures and then recall as many picture names as they could recall during 30 seconds. The last task is the Five-line test shown in Supplementary Figure 3 and the comparable Four-line test in parallel version 2 with similar results (Hollá & Bartoš, 2017). An examinee draws as many different shapes from five or four straight lines as possible in one minute. The shapes has not to be repeated, even not in other positions (rotated, mirror-inverted, etc). This subtest is focused on the executive, visual, visual-spatial functions, strategic and logical thinking (Bartoš & Raisová, 2019). In online testing an administrator asks an examinee to draw shapes from five/four lines on a paper and then shows it through a web camera.

**Evaluation and scoring of the Assessment BAttery of COgnition (ABACO)**

A total score of ABACO ranges from 0 (the worst) to 35 points (the best). It consists of these parts and scores: (1) the number of correctly read entire sentences in the Reading Encrypted Sentences subtest (score: 0–3 points), (2) the number of weighted scores derived from generated number of fruits or profession during 30 seconds (score: 0–6), (3) the weighted number of points for wrongly named pictures of the PICNIR (score: 0–2), (4) the number of points for correctly recalled pictures of the PICNIR test (score: 0–10), (5) the number of points for correctly drawn shapes in the Five or Four-line test (score: 0-4), (6) the number of correctly recalled words of the sentence during delayed recall (score: 0-10).

**References:**

Hollá, M., and Bartoš, A. (2017). Reliabilita paralelních forem nových kognitivních testů.

[dissertation/master’s thesis in Czech]. Masarykova Univerzita.

Bartoš, A., and Raisová, M. (2019). Testy a dotazníky pro vyšetřování kognitivních funkcí, nálady a soběstačnosti (2nd ed.). Praha: Mladá fronta.

Bartoš, A. (2018). Remember POBAV – a short test of picture naming and their recall aimed for

early detection of cognitive impairment [article in Czech Pamatujte na POBAV – krátký test

pojmenování obrázků a jejich vybavení sloužící ke včasnému záchytu kognitivních poruch]. *Neurol.* *praxi.* 19(Suppl.1), 5-10.

Bartoš A. (2016). Do not Test but POBAV (ENTERTAIN) – Written Intentional Nam ing of Pictures and their Recall as a Brief Cognitive Test [article in Czech Netestuj, ale POBAV: písemné záměrné Pojmenování OBrázků A jejich Vybavení jako krátká kognitivní zkouška]. *Cesk. Slov. Neurol*. 79/112, 671-679.
