# Supplementary figures and images for: Effect of human probiotics on memory, psychological and biological measures in elderly: A study protocol of bi-center, double-blind, randomized, placebo-controlled clinical trial (CleverAge Biota)
Source: Front Aging Neurosci. 2022 Nov 10;14:996234. doi: 10.3389/fnagi.2022.996234 (PMC9686296; doi:10.3389/fnagi.2022.996234)

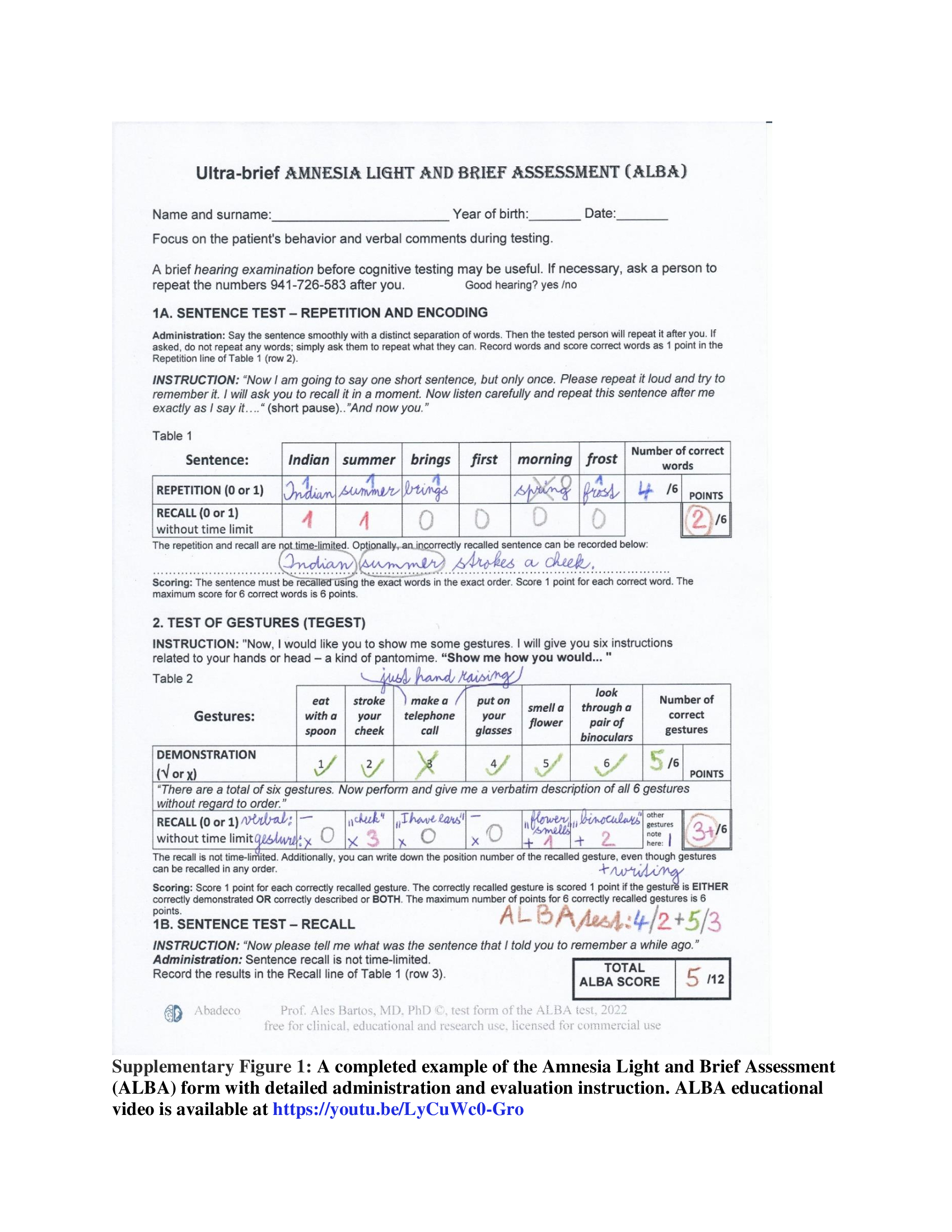

Supplement: Supplementary file 4 [file Image_1.JPEG]

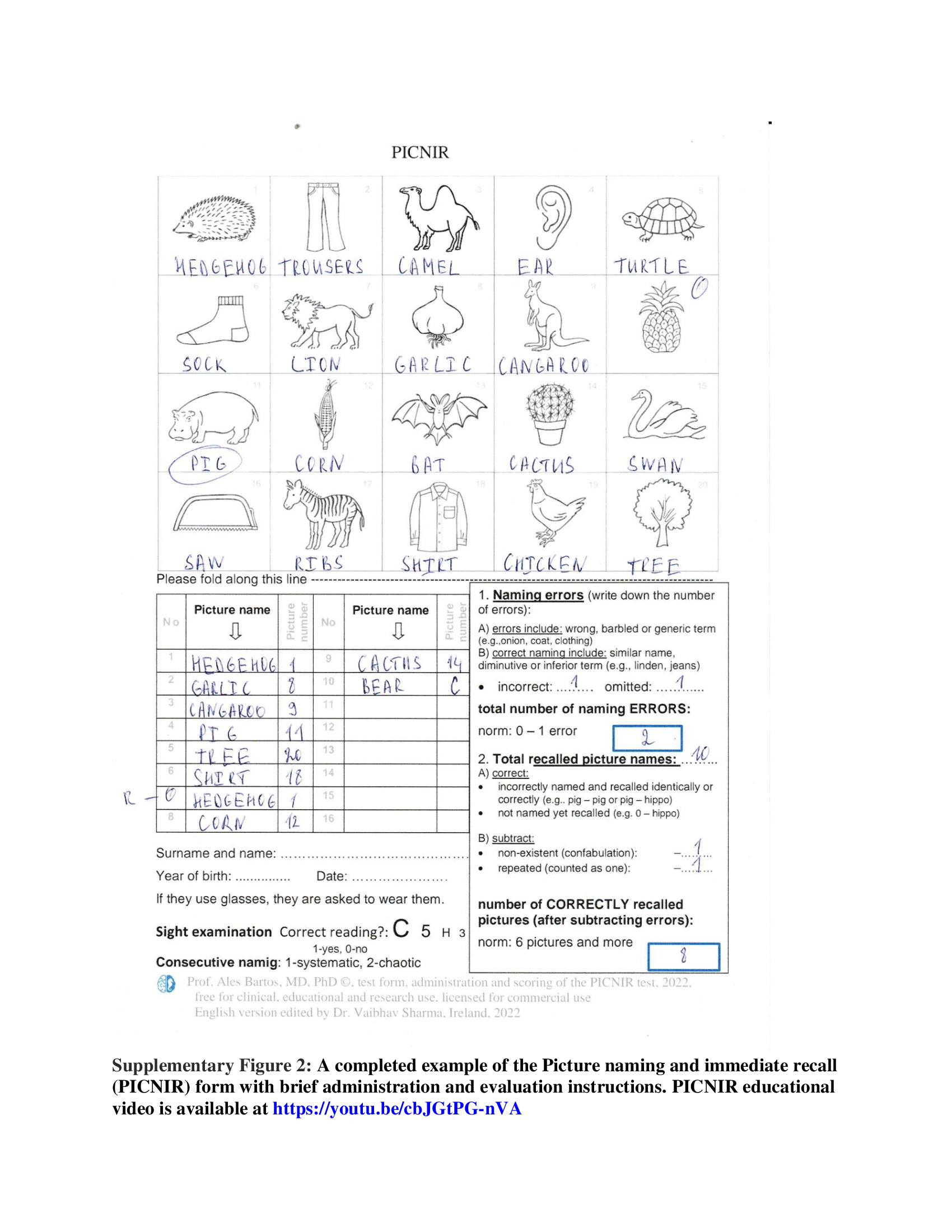

Supplement: Supplementary file 5 [file Image_2.JPEG]

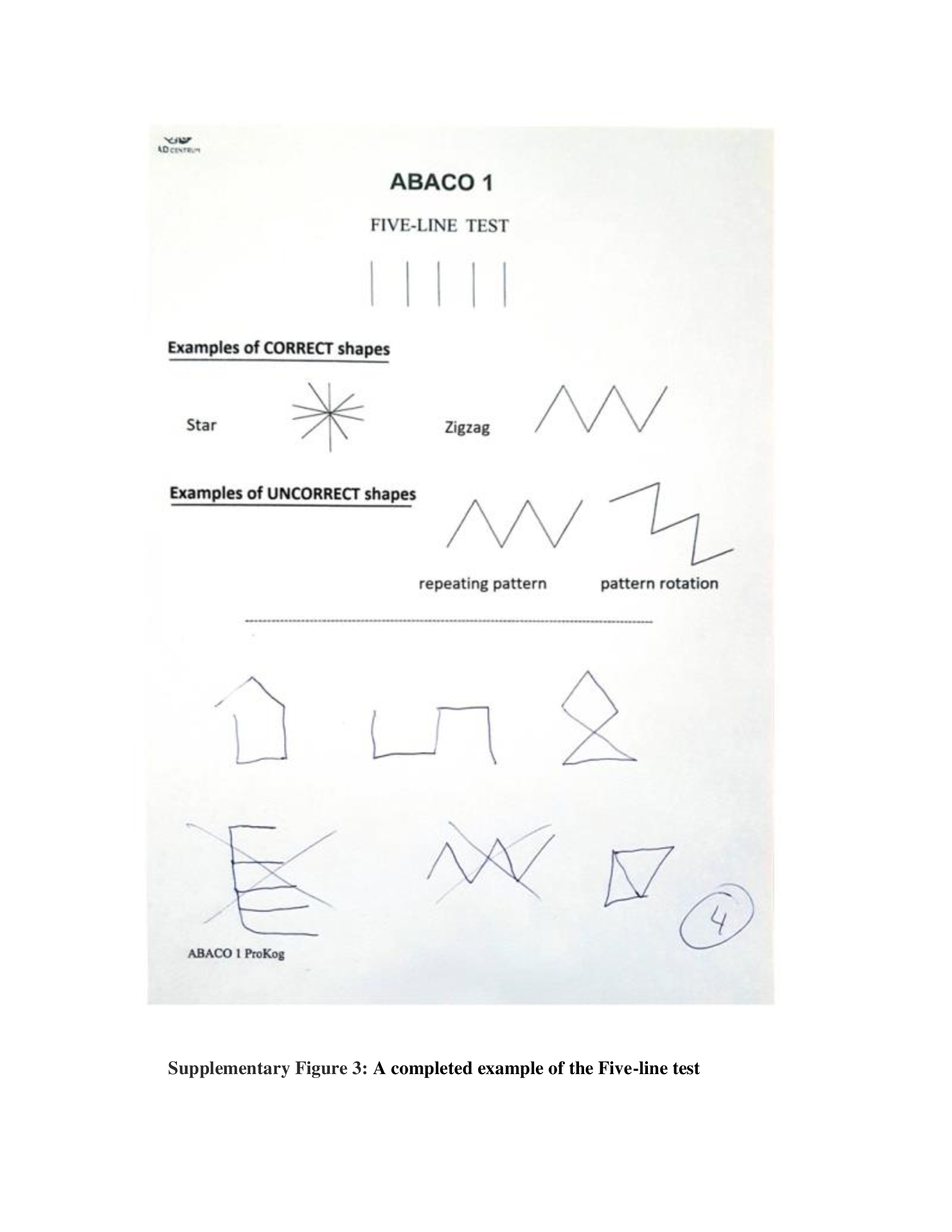

Supplement: Supplementary file 6 [file Image_3.JPEG]
